# Draft Genome Sequences of Four Vibrio parahaemolyticus Isolates from Clinical Cases in Canada

**DOI:** 10.1128/genomeA.01482-14

**Published:** 2015-01-29

**Authors:** Swapan Banerjee, Nicholas Petronella, Courtney Chew Leung, Jeffrey Farber

**Affiliations:** a Bureau of Microbial Hazards, Health Canada, Ottawa, Ontario, Canada; b Bureau of Food Surveillance and Science Integration, Health Canada, Ottawa, Ontario, Canada

## Abstract

Vibrio parahaemolyticus is a leading cause of bacterial gastroenteritis following ingestion of contaminated seafood. This report presents the draft genome sequences of four clinical strains of V. parahaemolyticus isolated in Canada. All four strains lack traditional pathogenic markers and possess uniquely individual characteristics identified using other typing criteria.

## GENOME ANNOUNCEMENT

Vibrio parahaemolyticus is a halophilic bacterium associated with plankton and seafood harvested from estuaries around the world. Some strains of V. parahaemolyticus are pathogenic and cause seafood-borne illnesses (1, 2). Pathogenicity is determined by confirming the presence of thermostable direct hemolysin (TDH) and TDH-related hemolysin (TRH) encoded by *tdh* and *trh*, respectively (1, 3, 4). The majority of clinical isolates test positive for one or both of these hemolysin genes by PCR. However, isolates lacking both *tdh* and *trh* markers have been detected in patients and reported in recent years from Canada (5) as well as from the United States (6). Here, we report the draft genomic sequences of four V. parahaemolyticus clinical isolates that lack the known virulence markers (*tdh*^−^ *trh*^−^). The strains were found to be diverse in terms of genotypic characteristics and geographic location (5). Genome sequences of V. parahaemolyticus strains, RIMD 2210633, *tdh*^+^ *trh*^−^ (7), and AQ4037, *tdh*^−^ *trh*^+^ (8), have been reported earlier and used as references for comparative analysis.

Short-read sequence data for the V. parahaemolyticus isolates were generated using a HiSeq Benchtop sequencer (Illumina, San Diego) for 500 cycles. Sequence data were assembled as described earlier (9, 10). The number of reads generated was 5,518,100 for strain T12739, 6,752,516 for T9109, 7,207,234 for 09-5357, and 3,732,814 for 04-2548. Reads were assembled *de novo* into high-quality draft genomes with SPAdes v3.1.1 (11, 12), resulting in 55 nonoverlapping contiguous sequences (contigs) with a total length of 5,114,934 bases, 45.29% G+C content, and 108-fold coverage for T12739. The assembly of T9109 resulted in 63 non-overlapping contigs with a total length of 5,082,583 bases, 45.30% G+C content, and 133-fold coverage. In addition, 09-5357 was assembled into 49 nonoverlapping contigs with a total length of 5,297,642 bases, 45.27% G+C content, and 136-fold coverage. Lastly, 04-2548 was assembled into 101 nonoverlapping contigs with a total length of 5,549,224 bases, 45.05% G+C content, and 68-fold coverage.

All four V. parahaemolyticus isolates tested positive by PCR for the presence of thermolabile hemolysin (tlh). Sequence comparison with the reference strains confirmed the absence of the known virulence markers (*tdh*, *trh*) in all four isolates. Preliminary sequence analysis targeting the two type III secretion systems (T3SSs) indicated the presence of the 11 core T3SS-1 related genes in all four isolates. In addition, the presence of transcriptional regulator *opaR* (VP2516) and adhesion factor *mam7* (VP1611) were confirmed in all four strains, providing evidence of some regulatory and host cell attachment functions (13). However, the sequences of 12 core proteins of T3SS-2 were not detected in any of the isolates reported here, suggesting that the complete virulence mechanism of these strains will require continued investigation.

### Nucleotide sequence accession numbers.

These whole-genome shotgun studies have been deposited at DDBL/EMBL/GenBank under accession numbers JTGS00000000 (04-2548), JTGQ00000000 (T12739), JTGR00000000 (T9109), and JTGT00000000 (09-5357). The first versions described in this announcement are JTGS01000000, JTGQ01000000, JTGR01000000, and JTGT01000000, respectively.
